# Regional Associations of Cortical Superficial Siderosis and β-Amyloid-Positron-Emission-Tomography Positivity in Patients With Cerebral Amyloid Angiopathy

**DOI:** 10.3389/fnagi.2021.786143

**Published:** 2022-02-03

**Authors:** Anika Finze, Hannes Wahl, Daniel Janowitz, Katharina Buerger, Jennifer Linn, Axel Rominger, Sophia Stöcklein, Peter Bartenstein, Frank Arne Wollenweber, Cihan Catak, Matthias Brendel

**Affiliations:** ^1^Department of Nuclear Medicine, University Hospital of Munich, LMU Munich, Munich, Germany; ^2^Department of Neuroradiology, University Hospital of Dresden, Carl Gustav Carus University Dresden, Dresden, Germany; ^3^Institute for Stroke and Dementia Research, University Hospital of Munich, LMU Munich, Munich, Germany; ^4^Department of Nuclear Medicine, Inselspital, Bern University Hospital, University of Bern, Bern, Switzerland; ^5^Department of Radiology, University Hospital of Munich, LMU Munich, Munich, Germany; ^6^Munich Cluster for Systems Neurology (SyNergy), Munich, Germany; ^7^German Center for Neurodegenerative Diseases (DZNE), Munich, Germany

**Keywords:** cerebral amyloid angiopathy, cortical superficial siderosis, β-amyloid, positron-emission-tomography, magnetic-resonance-imaging, topology, colocalization

## Abstract

**Objective:**

This is a cross-sectional study to evaluate whether β-amyloid-(Aβ)-PET positivity and cortical superficial siderosis (cSS) in patients with cerebral amyloid angiopathy (CAA) are regionally colocalized.

**Methods:**

Ten patients with probable or possible CAA (73.3 ± 10.9 years, 40% women) underwent MRI examination with a gradient-echo-T2*-weighted-imaging sequence to detect cSS and ^18^F-florbetaben PET examination to detect fibrillar Aβ. In all cortical regions of the Hammers Atlas, cSS positivity (MRI: ITK-SNAP segmentation) and Aβ-PET positivity (PET: ≥ mean value + 2 standard deviations of 14 healthy controls) were defined. Regional agreement of cSS- and Aβ-PET positivity was evaluated. Aβ-PET quantification was compared between cSS-positive and corresponding contralateral cSS-negative atlas regions. Furthermore, the Aβ-PET quantification of cSS-positive regions was evaluated in voxels close to cSS and in direct cSS voxels.

**Results:**

cSS- and Aβ-PET positivity did not indicate similarity of their regional patterns, despite a minor association between the frequency of Aβ-positive patients and the frequency of cSS-positive patients within individual regions (*r*_s_ = 0.277, *p* = 0.032). However, this association was driven by temporal regions lacking cSS- and Aβ-PET positivity. When analyzing all composite brain regions, Aβ-PET values in regions close to cSS were significantly higher than in regions directly affected with cSS (*p* < 0.0001). However, Aβ-PET values in regions close to cSS were not different when compared to corresponding contralateral cSS-negative regions (*p* = 0.603).

**Conclusion:**

In this cross-sectional study, cSS and Aβ-PET positivity did not show regional association in patients with CAA and deserve further exploitation in longitudinal designs. In clinical routine, a specific cross-sectional evaluation of Aβ-PET in cSS-positive regions is probably not useful for visual reading of Aβ-PETs in patients with CAA.

## Introduction

Cerebral amyloid angiopathy (CAA) is characterized by leptomeningeal and cortical beta-amyloid (Aβ) accumulation in small vessels (Vinters, 1987). CAA particularly affects the aged population, and it is associated with Alzheimer’s disease (AD; Ellis et al., 1996; Keage et al., 2009; Arvanitakis et al., 2011). Previous studies have indicated that CAA is characterized by vascular occlusion and local ischemia (Olichney et al., 1995; Cadavid et al., 2000; Thal et al., 2009). Besides, the local fragility of the vessels can result in singular or chronic intracerebral or subarachnoid bleeding (Vinters, 1987; Itoh et al., 1993; Qureshi et al., 2001, 2009; Nakata-Kudo et al., 2006; Charidimou et al., 2012). The typical CAA finding of linear cortical superficial siderosis (cSS) winds around the gyri and sulci of the brain and the blood component hemosiderin, in particular, can be measured in gradient-echo-T2*-weighted-imaging (GRE-T2*-WI), a sequence of MRI (Linn et al., 2008, 2010; Kumar, 2010). Recent studies pointed out a diagnostic (Linn et al., 2010; Charidimou et al., 2013a) and prognostic (Charidimou et al., 2013b; Linn et al., 2013; Wollenweber et al., 2019) value of cSS determination in patients with CAA. However, the detailed mechanisms and associations between Aβ aggregation and cSS are still unclear. The aforementioned findings suggest that there is a regional relationship between Aβ accumulation and cSS, and we aimed to test this hypothesis by *in vivo* imaging. In this regard, fibrillar Aβ is now sufficiently detectable by FDA-approved PET tracers in living patients (Clark et al., 2011; Curtis et al., 2015; Sabri et al., 2015), and modern data analysis pipelines allow to investigate the PET and MRI data in matched spatial orientation (Wollenweber et al., 2014). We hypothesized that regionally pronounced Aβ accumulation leads to a colocalized increase of cSS due to higher vessel vulnerability in these regions. In turn, this could facilitate improved judgment of Aβ-PET positivity by the specific analysis of cSS-positive brain regions. However, in a recent single-case analysis from our group (Brendel et al., 2020b), a patient with probable CAA did not show a regional increase of the Aβ-PET signal in brain areas affected by cSS. Thus, we aimed to explore this phenomenon in a larger cohort of ten patients with CAA who underwent GRE-T2*-WI MRI and ^18^F-florbetaben Aβ-PET. A detailed segmentation approach was used to investigate the regional agreement between fibrillar Aβ accumulation and cSS.

## Materials and Methods

### Participants

Ten CAA patients with cSS were investigated in this cross-sectional study. All have been diagnosed with a possible or probable CAA according to the modified Boston criteria (Linn et al., 2010). The presence of cSS was determined by experts using the GRE-T2*-WI MRI sequence. They underwent a standardized ^18^F-florbetaben PET examination (Barthel et al., 2011). Detailed inclusion and exclusion criteria and study protocols were published elsewhere (Wollenweber et al., 2014, 2019). Five patients were embedded in the Superficial Siderosis in Patients with Suspected Cerebral Amyloid Angiopathy study (SuSPect-CAA; Wollenweber et al., 2019), a prospective, multicenter cohort study (gov: NCT01856699). The other five patients were part of the longitudinal Determinants of Dementia after Stroke (DEDEMAS) cohort study (Wollenweber et al., 2014) (gov: NCT01334749). The data of one patient have already been published as a case report (Brendel et al., 2020b). As a control group [i.e., healthy control (HC)], 14 cognitively healthy individuals who underwent ^18^F-florbetaben PET with no evidence of CAA or cSS were included. They were cognitively inconspicuous and had no history of cerebral hemorrhage and a negative Aβ status to ^18^F-florbetaben PET (visual read by a single expert). HCs of the in-house database were excluded *a priori* if they showed a standardized uptake value ratio (SUVr) over 2.5 standard deviations (SDs) above the mean value (MV) of the other controls in one region to avoid controls at the early stages of Aβ buildup.

### Magnetic Resonance Imaging

The MRI examinations were performed on 3.0 Tesla GE Signa HDxt scanners. Since two different study protocols were used, the parameters differ in some cases. In this case, both parameters are given as follows: TE = 9 ms/14 ms, TR = 600 ms/540 ms, flip angle = 20°, slice thickness = 5 mm, spacing between slices = 5.5 mm, acquisition matrix = 320 × 320/256 × 256, reconstruction matrix = 512 × 512, inplane resolution = 0.43 × 0.43 mm/1 × 1 mm.

### ^18^F-Florbetaben Positron-Emission-Tomography Imaging

^18^F-florbetaben PET was carried out on a GE Discovery 690 PET/CT (GE Healthcare) or a Siemens Biograph 64 True X (Siemens, Erlangen, Germany) scanner with harmonized dedicated brain PET reconstruction protocols in terms of spatial resolution *via* Hofmann phantom measurements (Brendel et al., 2020a). After the intravenous injection of 300.9 ± 16.8 MBq ^18^F-florbetaben, a low-dose CT of the skull was performed for attenuation correction between 85 and 90 min, and a 20-min emission measurement was started 90 min post injection (Barthel et al., 2011).

### Image Data Processing

**FIGURE 1:**
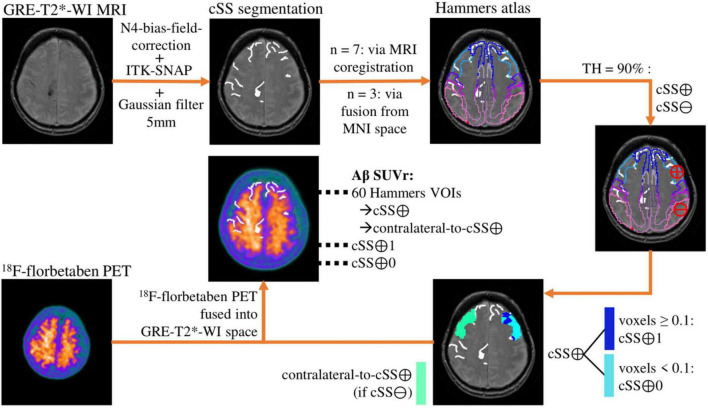
Methodological workflow. The analysis pipeline is illustrated by the images of an exemplary patient. The cortical superficial siderosis (cSS) segmentation is shown without the 5-mm Gaussian filter.

Figure 1 provides an overview of all methodological steps. First, the T2* sequences were homogenized with N4 bias field correction to minimize segmentation errors due to variances in field intensity (Tustison et al., 2010). Second, expert raters segmented the cSS-affected gyri and sulci in each axial T2* sequence using ITK-SNAP (V3.6) (Yushkevich et al., 2004), by defining cSS with a value of 1 and non-cSS with 0. A brush diameter of 6 mm was used. In the next step, the segmented cSS was smoothed using a 5-mm Gaussian filter to reflect the PET resolution. For each patient, the Hammers Atlas (Hammers et al., 2003, 2008; Gousias et al., 2008), consisting of 83 volumes-of-interests (VOIs), was adapted to the respective GRE-T2*-WI MRI. For seven patients, a three-dimensional (3D) T1-weighted MRI sequence was used to outline the brain into the 83 atlas VOIs using the PNEURO tool implemented by PMOD (V3.5, PMOD Technologies LLC, Zurich, Switzerland). The created VOI-set was then fused into the GRE-T2*-WI MRI of patients using the FUSION tool (V3.5, PMOD Technologies LLC). For the other three patients without 3D T1-weighted MRI, a Hammers VOI-set adapted to the Montreal Neurological Institute (MNI) space (Evans et al., 1993; Collins et al., 1994; Montreal Neurological Institute, 2012) was fused into the GRE-T2*-WI MRI. All subcortical VOIs were excluded for this analysis, reducing the VOI-set to 60 regions (30 in each hemisphere). Using a probability threshold of 90%, the previously adjusted and reduced 60 atlas VOIs were thus defined as cSS-positive (cSS⊕) or cSS-negative (cSS⊖). In a cSS⊕-VOI, all voxels with a value ≥0.1 were subdivided into cSS⊕-with-siderosis (cSS⊕1), and all voxels with a value <0.1 were subdivided into cSS⊕-without-siderosis (cSS⊕0). For cSS⊕-VOIs, the corresponding contralateral VOI was defined as a control region when it was cSS⊖ (contralateral-to-cSS⊕). Finally, the ^18^F-florbetaben PET was fused into the respective GRE-T2*-WI space, and Aβ SUV was determined from all 60 Hammers (including cSS⊕ and contralateral-to-cSS⊕), cSS⊕1, and cSS⊕0 VOIs. The left and right cerebellum (additional VOIs from Hammers Atlas) were averaged and used as a reference region, which served for the calculation of cortex-to-cerebellum SUVr. In summary, SUVr were determined in cSS⊕, cSS⊕1, cSS⊕0, and contralateral-to-cSS⊕ regions. The co-registered images of one patient were superimposed for a descriptive single-case presentation.

### Statistical Analysis

All statistical analyses were carried out with SPSS (V25, IBM, Ehningen, Germany) or R (V4.0.2, R Core Team, 2020, Vienna, Austria). The level of significance was set at α = 0.05. Age was determined by the date of the ^18^F-florbetaben PET acquisition. Age was compared between the study and control group by a two-tailed Student’s *t*-test, whereas sex was subjected to a χ^2^ test.

A semiquantitative region-based classification was performed in all cortical atlas regions (26 frontal, 20 temporal, six parietal, six occipital, and two posterior cingulate gyrus = 60 regions), defining Aβ-PET SUVr ≥ MV + 2.5 SD of the controls as Aβ-positive (Aβ⊕). Furthermore, the regions were divided into cSS-positive (cSS⊕) and cSS-negative (cSS⊖) with a probability threshold of 90%, as already described above. The frequency of Aβ-positive patients in an atlas region was correlated with the frequency of cSS-positive patients by calculating Spearman’s correlation coefficient (*r*_s_). The agreement of Aβ-PET positivity with cSS positivity of all single regions was assessed.

For the comparison of SUVr, multiple two-tailed pairwise Student’s *t*-tests between the four previously defined VOI groups were performed, to account for dependent samples. cSS⊕, cSS⊕1, and cSS⊕0 were compared with each other, as well as cSS⊕0 with the region contralateral to cSS⊕, if it was cSS⊖ (contralateral-to-cSS⊕). These quantitative group comparisons were carried out individually for all common Aβ-PET target regions [i.e., frontal, temporal, parietal, and posterior cingulate gyrus (Brendel et al., 2015)] and for the composite of these regions. To correct for multiple testing, the *p*-value adjustments were made within each lobe analysis (four *t*-tests in one lobe) using false-discovery-rate (FDR) correction (Benjamini and Hochberg, 1995). Since there is an additional risk of alpha error accumulation through statistical testing in five cerebral lobes, each *p*-value was multiplied by five according to the Bonferroni correction (Armstrong, 2014).

## Results

The patients presented themselves to the respective clinics with focal neurological symptoms (past intracranial hemorrhage *n* = 3, memory complaints *n* = 4, past ischemic stroke or transient ischemic attack *n* = 2, or presumed focal seizures *n* = 1). Of the four women (66.2 ± 14.4 years) and six men (78.0 ± 4.9 years), three patients were diagnosed with a possible CAA and seven patients with a probable CAA, according to the modified Boston criteria (Linn et al., 2010). HCs were not significantly different in age (patients: 73.3 ± 10.9 years; HC: 65.9 ± 5.7 years; *p* = 0.073) and sex (patients: M/F = 6/4; HC: M/F = 8/6; χ^2^ = 0.020; *p* = 0.889). Details of the cohort are provided in Table 1.

**TABLE 1:** Demographics and clinical data of patients and healthy controls (HCs) (same order as Figure 2).

Patient	CAA: possible = 0 probable = 1	cSS⊕ + Aβ⊕ (/cSS⊕) in %	cSS: focal = 0, disseminated = 1	Sex (♂1/♀0)	Age (birth-PET)	^18^F-florbetaben (MBq)	MMSE	Arterial hypertension = AH, Hypercholesterolemia = HC, Diabetes mellitus = DM
CAA 1	1	0%	1	1	74	291.15	28	None
CAA 2	1	81%	1	1	79	302.37	14	HC
CAA 3	0	100%	0	1	79	294.00	20	None
CAA 4	1	21%	1	0	75	303.00	6	HC
CAA 5	0	75%	0	0	70	280.00	26	AH
CAA 6	1	73%	1	1	85	299.00	29	HC
CAA 7	1	52%	1	1	71	288.34	26	AH
CAA 8	1	69%	1	0	45	295.77	21	None
CAA 9	1	100%	0	1	80	295.00	28	AH/HC/DM
CAA 10	0	0%	0	0	75	299.00	28	AH/HC/DM
Avg.	poss 3/prob 7	57.1 ± 37.8	f 4/d 6	♂6/♀4	73.3 ± 10.9	294.8 ± 7.09	22.6 ± 7.5	AH 4/HC 5/DM 2
CAA 1 + 10 + 4 + 7 + 8	poss 1/prob 4	<70%	f 1/d 4	♂2/♀3	68.0 ± 13.0	295.5 ± 5.9	21.8 ± 9.3	AH 2/HC 2/DM 1
CAA 6 + 5 + 2 + 3 + 9	poss 2/prob 3	>70%	f 3/d 2	♂4/♀1	78.6 ± 5.4	294.1 ± 8.5	23.4 ± 6.3	AH 2/HC 3/DM 1
Difference (<70% and >70%) *p*-value			χ^2^-test		Two-tailed Student’s *t*-tests			n.a.
			0.197	0.197	0.148	0.775	0.759	
HC 1	n.a.	n.a.	n.a.	0	63	293.00	n.a.	n.a.
HC 2	n.a.	n.a.	n.a.	1	60	309.00	n.a.	n.a.
HC 3	n.a.	n.a.	n.a.	1	66	299.00	n.a.	n.a.
HC 4	n.a.	n.a.	n.a.	1	58	315.00	n.a.	n.a.
HC 5	n.a.	n.a.	n.a.	0	65	315.00	n.a.	n.a.
HC 6	n.a.	n.a.	n.a.	1	65	301.00	n.a.	n.a.
HC 7	n.a.	n.a.	n.a.	0	69	326.00	n.a.	n.a.
HC 8	n.a.	n.a.	n.a.	1	70	297.00	n.a.	n.a.
HC 9	n.a.	n.a.	n.a.	0	66	271.00	n.a.	n.a.
HC 10	n.a.	n.a.	n.a.	1	66	293.00	n.a.	n.a.
HC 11	n.a.	n.a.	n.a.	0	64	352.00	n.a.	n.a.
HC 12	n.a.	n.a.	n.a.	1	72	322.00	n.a.	n.a.
HC 13	n.a.	n.a.	n.a.	0	59	288.00	n.a.	n.a.
HC 14	n.a.	n.a.	n.a.	1	80	295.00	n.a.	n.a.
avg.	n.a.	n.a.	n.a.	♂8/♀6	65.9 ± 5.7	305.4 ± 19.8	n.a.	n.a.
Difference (CAA and HC) *p*-value			n.a.	X^2^-test	two-tailed Student’s *t*-tests		n.a.	n.a.
			n.a.	0.889	0.073	0.080	n.a.	n.a.

*For patients with cerebral amyloid angiopathy (CAA), the data were split into two subgroups, which was performed with five patients each to test for group differences of patients with less and patients with more β-amyloid (Aβ) and cortical superficial siderosis (cSS) overlap.*

**FIGURE 2:**
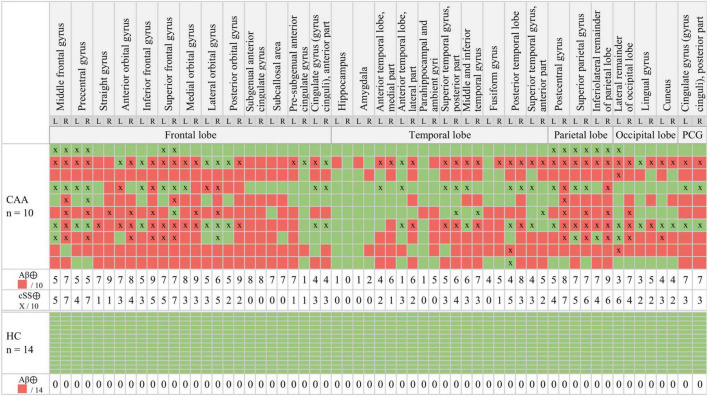
Multiregion classifier. β-amyloid (Aβ): The semiquantitative classification of each brain region (red = Aβ⊕, green = Aβ⊖) was performed by applying a mean value (MV) + 2.5 standard deviations (SDs) threshold obtained from healthy controls (HCs). Cortical superficial siderosis (cSS): cSS⊕ was defined with a probability threshold of 90% (*x* = cSS⊕).

**FIGURE 3:**
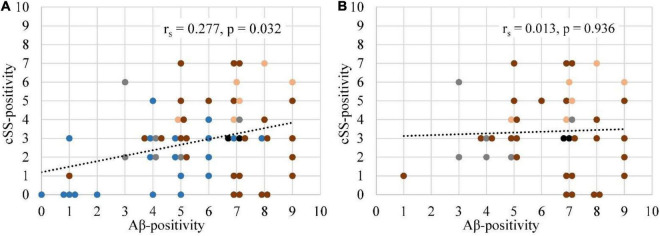
Association between the frequencies of Aβ-PET positivity and cSS positivity. **(A)** The frequency of Aβ-positive (SUVr ≥ 2.5 SD above the MV of the HCs) patients was correlated with the frequency of cSS-positive (90% probability threshold) patients for all 60 regions. Details are provided in Figure 2. **(B)** A repeated analysis was performed with the exclusion of temporal regions. The degree of association was calculated by Spearman’s correlation coefficient (*r*_s_). If two or more points had the same coordinates, they were slightly scattered in a horizontal plane. Each brain lobe was assigned its own color: 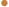 Frontal lobe 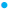 Temporal lobe 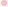 Parietal lobe 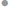 Occipital lobe 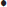 Posterior cingulate gyrus.

**FIGURE 4:**
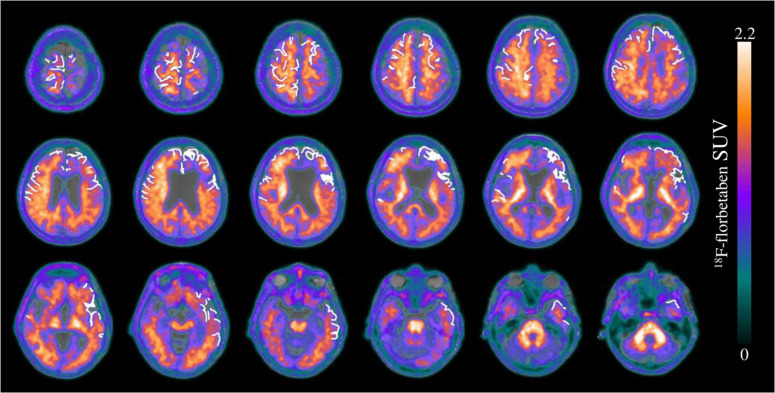
Transverse brain sections of an exemplary patient with cerebral amyloid angiopathy (CAA) (female, 75 years). The superimposition of cSS segmentation (white 0 and 1) and Aβ-PET (SUVr = 0–2.2) on a structural T1 magnetization prepared rapid gradient echo (MPRAGE) MRI sequence. The segmented cSS winded around the gyri and sulci as a linear structure. Since the patient was characterized by more than three affected sulci, cSS was classified as disseminated. A slight right-sided dominance was observed for the Aβ-PET signal, whereas the cSS distribution was left-sided predominantly in the frontal and the temporal lobe.

According to the multiregion classifier (Figure 2), nine patients (9/10 = 90%) showed at least one, and all patients showed an average of 37.0 ± 12.6 Aβ-PET-positive brain regions. Four patients had a focal cSS (≤3 sulci) with a mean of 2.0 ± 1.4 cSS⊕ regions, and the other six patients had a disseminated cSS (>3 sulci) with a mean of 27.0 ± 14.8 cSS⊕ regions (Linn et al., 2010). The patient without any Aβ-positive region was rated with a disseminated cSS on MRI with 13 cSS-positive regions. From a total of 170 cSS⊕ regions in all 10 patients, 40 were observed unilateral, whereas the remaining 130 were observed bilateral (65 in each hemisphere). The frequency of cSS- and Aβ-positive patients (as shown in Figure 2) for each region is plotted in Figure 3A, and Spearman’s Rho analysis showed a slightly significant positive correlation (*r*_s_ = 0.277, *p* = 0.032). However, this association was driven by temporal regions characterized by both limited cSS and Aβ-PET positivity (Figure 3B). As shown in Table 2, concurrent cSS- and Aβ-positive atlas regions (94/600 = 15.7%) did not occur more frequently than statistical random distribution. There were no significant differences in patient characteristics among patients with high and low cSS- and Aβ-positive region overlap (Table 1). Transverse brain sections of one CAA patient (female, 75 years) are illustrated in Figure 4.

**TABLE 2:** Regional Aβ-PET and cSS concordance.

		^18^F-florbetaben PET		
		Aβ⊕	Aβ⊖	
T2* MRI	cSS⊕	94	76	170
	cSS⊖	239	191	430
		333	267	600

*Aβ-PET and cSS status of all single brain regions, as classified by the multiregion classifier (Figure 2), and their respective agreement or disagreement. Each brain region of all ten patients was considered individually.*

**FIGURE 5:**
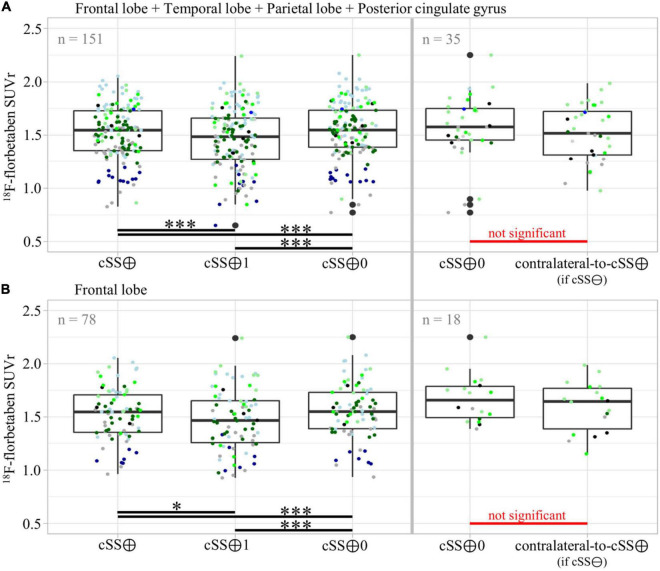
Group differences of Aβ-PET quantification dependent on vicinity to cSS. Pairwise group differences of Aβ-PET SUVr between cSS⊕, cSS⊕1, and cSS⊕0 regions, as well as between cSS⊕0 and contralateral-to-cSS⊕ (if cSS⊖) regions. In (A) all composite cerebral lobes were taken into account, and in (B) the frontal lobe was considered separately. Boxes represent the median, 25th, and 75th percentile. Each of the ten patients was assigned its own color. The descriptive data and respective statistics are listed in Tables 3A,B. Significance levels are indicated by *p < 0.05, ***p < 0.001.

To substantiate the descriptive results, a detailed quantitative approach was pursued to investigate the regional relationship between cSS and Aβ deposition in more detail. In this study, we tested the SUVr differences between the VOI groups defined earlier. All SUVr MVs and SDs are provided in Table 3A, whereas all *p*-values of the two-tailed pairwise Student’s *t*-tests are provided in Table 3B. The results of all composite cerebral lobes (Figure 5A) and the frontal lobe (Figure 5B) are illustrated separately in a boxplot. When analyzing all composite cerebral lobes (*n* = 151 regions), the group of cSS⊕1 regions revealed significantly lower Aβ SUVr when compared to cSS⊕0 regions (cSS⊕1: 1.462 ± 0.293; cSS⊕0: 1.538 ± 0.265; *p* < 0.0001), indicating that the tracer uptake was higher in the surrounding brain region of cSS when compared to the direct localization of cSS. This was also reflected by lower SUVr of cSS⊕1 regions when compared to combined cSS⊕ regions (cSS⊕1: 1.462 ± 0.293; cSS⊕: 1.517 ± 0.254; *p* < 0.001). Thus, we questioned if Aβ SUVr in the vicinity of cSS-positive regions indicates the hypothesized Aβ accumulation due to higher vessel vulnerability. However, we did not find an elevated Aβ SUVr in cSS⊕0 regions when compared to contralateral regions, if they were cSS-negative (= contralateral-to-cSS⊕) (*n* = 35; cSS⊕0: 1.568 ± 0.300; contralateral-to-cSS⊕: 1.515 ± 0.260; *p* = 0.603).

**TABLE 3:** ^18^F-florbetaben PET quantification at group level: **(A)** Values represent group means (MV) of ^18^F-florbetaben PET SUVr and their SD and **(B)** the *p*-values were derived from two-tailed pairwise Student’s *t*-tests with false-discovery-rate (FDR) and Bonferroni correction.

A	^18^F-florbetaben PET SUVr						
	*n*	cSS⊕	cSS⊕1	cSS⊕0	*n*	cSS⊕0	Contralateral-to-cSS⊕ (CL)
Composite lobes	151	1.517 ± 0.254	1.462 ± 0.293	1.538 ± 0.265	35	1.568 ± 0.300	1.515 ± 0.260
Frontal lobe	78	1.525 ± 0.246	1.466 ± 0.287	1.557 ± 0.258	18	1.673 ± 0.221	1.594 ± 0.236
Temporal lobe	35	1.475 ± 0.268	1.426 ± 0.234	1.498 ± 0.288	11	1.345 ± 0.368	1.314 ± 0.217
Parietal lobe	32	1.522 ± 0.269	1.462 ± 0.361	1.516 ± 0.267	6	1.659 ± 0.134	1.647 ± 0.212
PCG	6	1.633 ± 0.218	1.618 ± 0.287	1.626 ± 0.214	0	n.a.	n.a.

**B**		***p*-values**					
		***n***	**cSS⊕**	**cSS⊕**	**cSS⊕1**	***n***	**cSS⊕0**
			**cSS⊕1**	**cSS⊕0**	**cSS⊕0**		**CL**

Composite lobes		151	0.000	0.000	0.000	35	0.603
Frontal lobe		78	0.011	0.001	0.001	18	0.226
Temporal lobe		35	0.409	0.449	0.317	11	1.000
Parietal lobe		32	0.891	1.000	0.944	6	1.000
PCG		6	1.000	1.000	1.000	0	n.a.

*Subanalysis to the right in A and B compares only cSS⊕0 against contralateral-to-cSS⊕ regions (CL) when CL was lacking cSS.*

We asked if significant effects could be masked by a combination of regions deriving from different lobes. However, the separate analysis of the frontal lobe (*n* = 78) still revealed lower Aβ SUVr in cSS⊕1 than in cSS⊕0 regions (cSS⊕1: 1.466 ± 0.287; cSS⊕0: 1.557 ± 0.258; *p* < 0.001), and these Aβ SUVr were lower than in combined cSS⊕ regions (cSS⊕1: 1.466 ± 0.287; cSS⊕: 1.525 ± 0.246; *p* < 0.05). We did not find an elevated Aβ SUVr in cSS⊕0 frontal regions when compared to contralateral-to-cSS⊕ regions (*n* = 18; cSS⊕0: 1.673 ± 0.221; contralateral-to-cSS⊕: 1.594 ± 0.236; *p* = 0.226). A stand-alone analysis of the temporal lobe, parietal lobe, and posterior cingulate gyrus (PCG) displayed no significant differences between any VOI groups after FDR and Bonferroni correction.

## Discussion

Pathogenetic considerations suggest that Aβ accumulation and cSS could have a regional association, and the aim of this study was to evaluate this potential linkage in a cohort of ten CAA patients with cSS pathology and Aβ-PET imaging. We observed that regional Aβ-PET positivity correlated with regional cSS positivity in 60 predefined brain regions. However, concomitant Aβ-PET- and cSS-positive regions did not occur more frequently than expected from a statistical random distribution. Using detailed quantitative segmentation approaches, we explored that brain regions affected by cSS showed decreased Aβ-PET SUVr compared to brain areas in the vicinity to cSS. However, regions in the vicinity to cSS did not show altered Aβ-PET SUVr compared to cSS-negative contralateral brain regions.

For a proper interpretation of our results, the small sample size needs to be considered in detail and as a limitation. One out of the ten patients did not show any Aβ-positive brain region (Figure 2). We used a clear Aβ-negative control cohort with *a priori* exclusion of outliers in the single regions to ensure sensitivity for Aβ-PET positivity in the single regions of patients with CAA. Thus, we assumed that this single case did not have significant Aβ deposition in the brain. However, all patients were diagnosed with possible or probable CAA according to the modified Boston criteria (Linn et al., 2010), and a positivity rate of 90% fits to previous studies reporting Aβ positivity in patients with probable CAA between 60 and 100% (Jang et al., 2019). The prevalence of CAA increases with age and is associated with dementia syndromes such as AD but often remains asymptomatic until death (Ellis et al., 1996; Keage et al., 2009; Arvanitakis et al., 2011). The age distribution of our study cohort was consistent with the typical age of patients with CAA (Arvanitakis et al., 2011) and was slightly higher than our control group. Only one patient had a CAA atypical age of only 45 years, so a hereditary cause could be considered (O’donnell et al., 2000; Knudsen et al., 2001; Linn et al., 2010). However, this case was not the one without Aβ-positive regions.

As apparent by the analysis of the multiregion classifier (Figure 2), Aβ and cSS affect all cerebral brain lobes. The temporal lobe was less affected by Aβ deposition, which is consistent with the previous literature discussing the occipital lobe as the main CAA localization (Vinters and Gilbert, 1983; Vinters, 1987; Attems et al., 2007, 2011). The cSS of our sample was represented strongest in the parietal lobe and least represented in the temporal lobe. Larger studies refer to the occipital (Shams et al., 2016) or frontal lobe (Zonneveld et al., 2014) as the most cSS-affected lobe, and the temporal lobe, as the less cSS-affected lobe (Zonneveld et al., 2014). Accordingly, previous studies vary as to the main localization of Aβ and cSS, but our sample was in line with the literature. We calculated Spearman’s correlation between the frequency of Aβ-positive patients and the frequency of cSS-positive patients in all 60 atlas regions, showing a slightly positive trend of colocalization (Figure 3). In this study, the temporal lobe showed regions with both low Aβ and low cSS, among them hippocampus and amygdala. Thus, the borderline positive correlation was likely driven by the low positivity rates of both biomarkers in the temporal lobe. This was confirmed by an analysis excluding the temporal lobe, which did not reveal significant colocalization. For an in-depth analysis, we investigated Aβ-PET positivity and cSS in all 600 regions to determine whether Aβ- and cSS positivity are colocalized (Table 2). Since 333 of the 600 regions were Aβ-positive (333/600 = 55.5%) and 170 were cSS-positive (170/600 = 28.3%), we expected 94.4 regions to be both cSS- and Aβ-positive in a statistical random distribution. With 94 out of 600 regions comprising combined cSS- and Aβ positivity, we did not find evidence for a non-random distribution. Similarly, the visual inspection of single cases (Figure 4) did not indicate that increased Aβ deposits are present in direct or immediate vicinity to cSS deposition. This cohort-based finding confirmed the previous case report of one patient from this study (Brendel et al., 2020b).

The atlas-based analysis provided a robust descriptive analysis of cSS and Aβ pathology distribution, but we also applied a methodologically more sophisticated approach to increase sensitivity for colocalization by segmenting the atlas regions. Therefore, we subsegmented all cSS-positive atlas regions (cSS⊕) into a part with only cSS (cSS⊕1) and a part surrounding the cSS but excluding direct cSS (cSS⊕0). The respective contralateral atlas regions were used as a reference group once they were cSS-negative (contralateral-to-cSS⊕). When analyzing all composite brain lobes (Figure 5A) and the frontal lobe separately (Figure 5B), less Aβ-PET signal was observed in regions with only cSS compared to composed atlas regions and compared to cSS surroundings. These group differences were proved to be significant despite rigorous correction for multiple testing. Among other factors, areas with cSS deposits may experience remodeling and structural changes in the brain parenchyma and cortical layers. The structural remodeling also alters the vascular supply and blood flow in the tissue; vessels may recede or be remodeled. This implies that the reduced vascular supply or the reduced cerebral blood flow could underlie the observed SUVr that decreases, since this semiquantitative measure of PET does not account for the alterations of perfusion changes (Berckel et al., 2013). Due to the accumulation of toxic species, not only changes in vascular structures but also neuronal loss and brain atrophy are expected to occur in the vicinity to cSS. In this regard, we did not account for partial-volume-effects (PVE) due to atrophy since our segmentation approach did not fit into available PVE correction models (Brendel et al., 2015). Hence, signal loss due to atrophy could be another confounder of the observed decreased SUVr. Then, the altered plaque morphology in terms of reduced Aβ plaque density could also lead to a reduced signal in PET (Biechele et al., 2021). In this regard, we noted that diffuse and vascular Aβ occurring at cSS sites provides fewer tracer binding sites when compared to cored Aβ plaques which could, in turn, hamper the detection by PET. Further studies with larger cohorts, a longitudinal study design, and complementary immunohistochemical examinations would be desirable to validate our results. Longitudinal studies will be of particular interest to better understand the mechanisms linking the occurrence of amyloid-related imaging abnormalities (ARIA) occurring both spontaneously in CAA-related inflammation with high levels of anti-amyloid autoantibodies (Antolini et al., 2021) and after exposure with monoclonal antibodies against plaque-deposited Aβ of AD immunotherapy (Chantran et al., 2019).

Our results are consistent with recent studies of methodologically different approaches that do not identify Aβ as a key mediator of neurodegeneration after stroke (Hagberg et al., 2020) or do not detect increased Aβ levels in PET in patients with pathologically confirmed CAA burden at the lobar level (McCarter et al., 2021). However, a large-scale population-based study identified a positive association between lobar cerebral microbleeds and Aβ load as assessed by PET, whereas there was no association between deep cerebral microbleeds and Aβ accumulation (Graff-Radford et al., 2019). Furthermore, a recent neuropathological investigation identified associations between advanced CAA in leptomeningeal vessels and cSS (Charidimou et al., 2020). Thus, vascular vulnerability, in general, has associations with Aβ accumulation, but cSS, in particular, does not further increase the regional probability of an elevated Aβ load. The clinical goal of this study was to determine whether there is a diagnostic benefit to look specifically at cSS-affected brain regions when classifying a patient as visually Aβ-positive or -negative. The results of our cohort study suggest that sensitivity for Aβ-PET positivity cannot be increased by considering cSS information from MRI.

## Conclusion

The colocalization of Aβ and cSS was not detectable with commonly used PET and MRI methodology *in vivo* in a small sample of patients with CAA. Therefore, the specific visual judgment of Aβ-PET positivity in cSS-positive regions is probably not a promising approach for the classification of Aβ-PET in patients with CAA.

## Data Availability Statement

The raw data supporting the conclusions of this article will be made available by the authors, without undue reservation.

## Ethics Statement

The studies involving human participants were reviewed and approved by the Ethics Committee of the Medical Faculty of the Ludwig Maximilian University of Munich. Written informed consent for participation was not required for this study in accordance with the national legislation and the institutional requirements.

## Author Contributions

AF contributed to writing, data analyses, and statistical analyses. SS, HW, and JL contributed to MRI data analyses. DJ, KB, FW, and CC contributed to patient recruitment, patient evaluation, and data analyses. AF, AR, PB, and MB contributed to PET scans and PET data analyses. FW, CC, and MB contributed to study design and conception. AF, CC, and MB contributed to writing, drafting, and manuscript conception. All authors added significant scientific input and intellectual content to the manuscript.

## Conflict of Interest

MB received speaker honoraria from GE Healthcare, Roche and LMI and is an advisor of LMI. AR and PB received speaker honoraria from GE Healthcare. JL received speaker honoraria from Bayer Vital GmbH and is an advisory board member of Mediaire GmbH and Biogen GmbH. The remaining authors declare that the research was conducted in the absence of any commercial or financial relationships that could be construed as a potential conflict of interest.

## Publisher’s Note

All claims expressed in this article are solely those of the authors and do not necessarily represent those of their affiliated organizations, or those of the publisher, the editors and the reviewers. Any product that may be evaluated in this article, or claim that may be made by its manufacturer, is not guaranteed or endorsed by the publisher.

## Abbreviations

CAA: cerebral amyloid angiopathy
cSS: cortical superficial siderosis
Aβ: β-amyloid
GRE-T2*-WI: gradient-echo-T2*-weighted-imaging
VOI: volume-of-interest
PVE: partial-volume-effects
SUVr: standardized-uptake-value-(ratio)
FDR: false-discovery-rate
HCs: healthy controls
SD: standard deviation
TH: threshold
MV: mean value
MNI: Montreal Neurological Institute
NA: not available
PCG: posterior cingulate gyrus.
